# Case report of a giant cervical leiomyoma

**DOI:** 10.3389/fmed.2025.1521984

**Published:** 2025-04-02

**Authors:** Fang Chen, Feng Bai, Yang Wang, Wenjuan Li

**Affiliations:** ^1^Department of Gynecology, People’s Hospital of Liaoning, People’s Hospital of China Medical University, Shenyang, China; ^2^Department of Gynecology, Liaoning Cancer Hospital and Institute, Cancer Hospital of China Medical University, Shenyang, China

**Keywords:** uterine leiomyoma, cervical leiomyoma, giant cervical leiomyoma, total hysterectomy, ureteral stent placement

## Abstract

This article reports the clinical diagnosis and treatment of a 42-year-old female patient with a giant cervical leiomyoma, a rare condition. A review of the literature over the past 30 years reveals only one reported case of a cervical interstitial leiomyoma larger than 20 cm. The surgical removal of such a large cervical smooth muscle tumor poses significant challenges. Due to the cervical location deep within the pelvis and its proximity to several vital organs, the presence of a giant tumor increases the risk of intraoperative bleeding and potential injury to adjacent structures. This paper systematically discusses the diagnostic and therapeutic approach for this patient, aiming to share our experience and review relevant literature, with the hope of providing reference for clinical treatment strategies.

## 1 Introduction

Uterine leiomyomas, commonly known as uterine fibroids, are prevalent benign tumors in gynecology, with an incidence ranging from 20 to 40% (1). Approximately 95% of these tumors arise in the uterine corpus (2), while cervical leiomyomas are exceedingly rare, occurring at a rate of only 0.6% (3). Among cervical leiomyomas, large cervical tumors are particularly uncommon. Cervical leiomyomas arise from cervical stromal tissue or vascular smooth muscle and can be classified based on their location into three categories: submucosal tumors within the cervical canal, intramural tumors, and paracervical tumors. They are typically solitary lesions (4). The precise etiology of uterine leiomyomas remains unclear; however, research suggests that elevated estrogen levels and prolonged estrogen stimulation may be contributing factors. Consequently, these tumors are more frequently observed in premenopausal women, with a tendency to regress post-menopause (5). Cervical leiomyomas, particularly those located in the lower cervix, often present as small masses in the early stages and may not be closely associated with surrounding organs, resulting in subtle clinical symptoms and challenges in early detection. When symptoms do arise, the tumors are usually significantly enlarged and may compress pelvic structures and nerves, leading to symptoms such as lower abdominal pain and discomfort in the lumbar region. Anterior or posterior growth of the tumors may exert pressure on the bladder, urethra, or rectum, potentially causing urinary frequency, difficulties in urination, urinary retention, or constipation. Lateral growth can compress the ureters, resulting in hydronephrosis, while pressure on pelvic vessels and lymphatics may lead to lower limb edema (6).

Despite the rarity of large cervical leiomyomas, systematic clinical studies focusing on symptomatic patients are currently lacking. Surgical resection remains the primary treatment approach for symptomatic large cervical smooth muscle tumors. However, due to the anatomical proximity of the cervix to the pelvis and other vital organs, the displacement caused by large leiomyomas complicates surgical procedures, significantly increasing operative difficulty and risk.

## 2 Case presentation

### 2.1 Clinical data

#### 2.1.1 Clinical history

A 42-year-old female was admitted with the chief complaint of “discovering uterine fibroids for 3 years and self-palpating an abdominal mass for 20 days.” Three years prior, a transvaginal ultrasound performed during a physical examination at an external hospital suggested “uterine fibroids” (size and location unspecified, report not provided). Over the past 2 years, the patient experienced an increase in menstrual volume, approximately double the usual amount, although the menstrual cycle and duration remained unchanged. Twenty days ago, the patient noted a hard mass in the lower abdomen and sought medical attention at our hospital, where a transvaginal ultrasound indicated multiple uterine fibroids, with the largest measuring 20 × 17 × 10 cm located in the cervix. One week ago, a pelvic MRI revealed multiple uterine fibroids and a large posterior cervical mass measuring 19.8 × 19.7 × 17.2 cm, with the bladder displaced anteriorly and downward. Additionally, there was dilation of the left renal pelvis and ureter, and hemoglobin levels were recorded at 56 g/L. The patient received intermittent red blood cell transfusions (4 units) to correct anemia, and follow-up blood tests showed hemoglobin at 88 g/L. She was admitted for further surgical intervention.

#### 2.1.2 Physical examination

**FIGURE 1:**
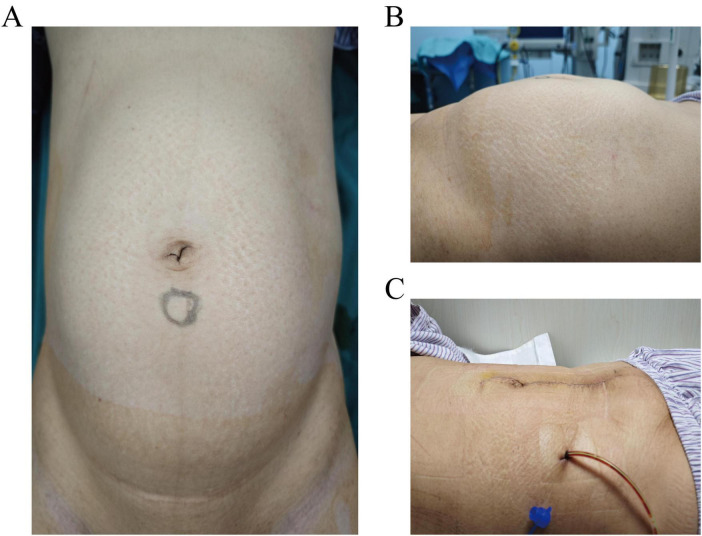
**(A,B)** Preoperative abdominal distension. **(A)** Anteroposterior view showing the upper border of the mass reaching three fingerbreadths above the umbilicus. **(B)** Lateral view demonstrating the mass extending laterally to the midaxillary line. **(C)** Postoperative day 2, incision dressing shows a flat abdomen.

The abdomen was soft, with no tenderness or rebound tenderness. A hard mass was palpated in the lower abdomen, reaching three fingerbreadths above the umbilicus and extending laterally to the midaxillary line (Figures 1A, B). Gynecological examination: The external genitalia were normal, the vagina was clear, and a small amount of white discharge was noted. The cervical anatomy could not be fully visualized; however, a hard mass was palpable in the posterior vaginal fornix, with its upper border located three fingerbreadths above the umbilicus. The uterine shape was not discernible, and no significant abnormalities were detected in the bilateral adnexal regions.

#### 2.1.3 Auxiliary examinations

Transvaginal Ultrasound: The uterus is anteverted, with an endometrial thickness of approximately 0.5 cm. Multiple hypoechoic lesions are observed in the myometrium, with the largest located in the cervix, measuring approximately 20 × 17 × 10 cm. Impression: Multiple uterine fibroids (intramural and subserosal).

**FIGURE 2:**
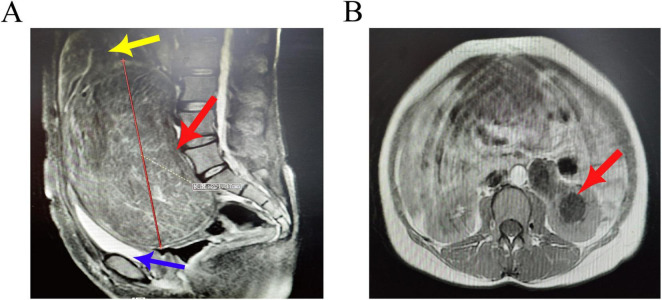
**(A)** Sagittal view of the pelvic MRI shows a large mass posterior to the cervix with well-defined margins, measuring approximately 20 cm. Multiple rounded short T2 signal lesions are observed in the myometrium, exhibiting mild enhancement (red arrow). The uterine body is elevated due to pressure, and there is elongation of the cervical canal (yellow arrow). The bladder is compressed and displaced anteriorly and downward (blue arrow). **(B)** Axial view demonstrates dilation of the left renal pelvis and ureter (red arrow).

Pelvic MRI: A large mass is observed posterior to the cervix, with well-defined margins, measuring approximately 19.8 × 19.7 × 17.2 cm. The uterine body is elevated due to pressure, and there is elongation of the cervical canal. Multiple rounded short T2 signal lesions are noted in the myometrium, exhibiting mild enhancement. The bladder is compressed and displaced anteriorly and downward (Figure 2A). The left renal pelvis and ureter are dilated (Figure 2B), with the distal segment of the left ureter showing unclear visualization. Some bowel segments in the pelvis are indistinct from the mass. Impression: A large space-occupying lesion posterior to the cervix, with a high likelihood of fibroids, indicating low-grade obstruction of the left urinary tract, multiple uterine fibroids, and heterogeneous signal in the uterine fundus and anterior wall, raising suspicion for adenomyosis.

### 2.2 Treatment methods

**FIGURE 3:**
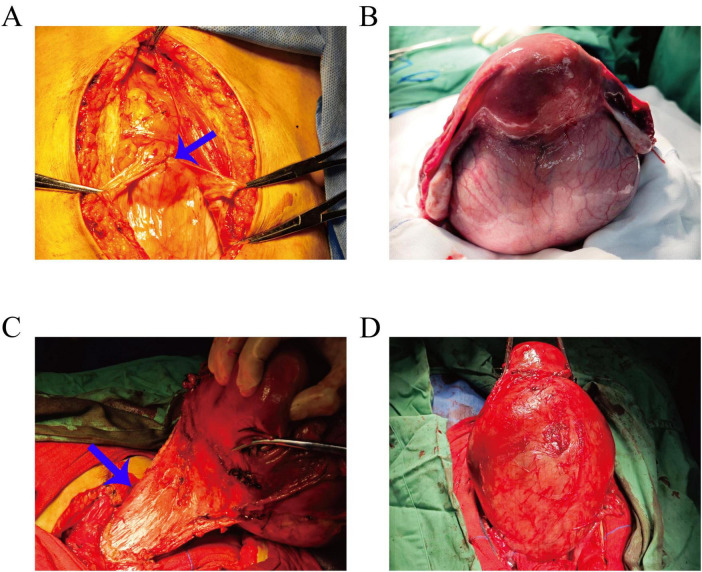
**(A)** Intraoperative view showing the bladder elevated and located between the umbilicus and the pubis (blue arrow). **(B)** The uterus is retracted, revealing an enlarged uterine body with a large cervical myoma extending downward from the cervical isthmus; numerous engorged blood vessels are visible on the myoma’s surface. **(C)** Intraoperative image demonstrating the cervical structure being significantly elongated (blue arrow). **(D)** After separation of the capsule, the large cervical myoma, approximately 20 cm in diameter, is being excised.

**FIGURE 4:**
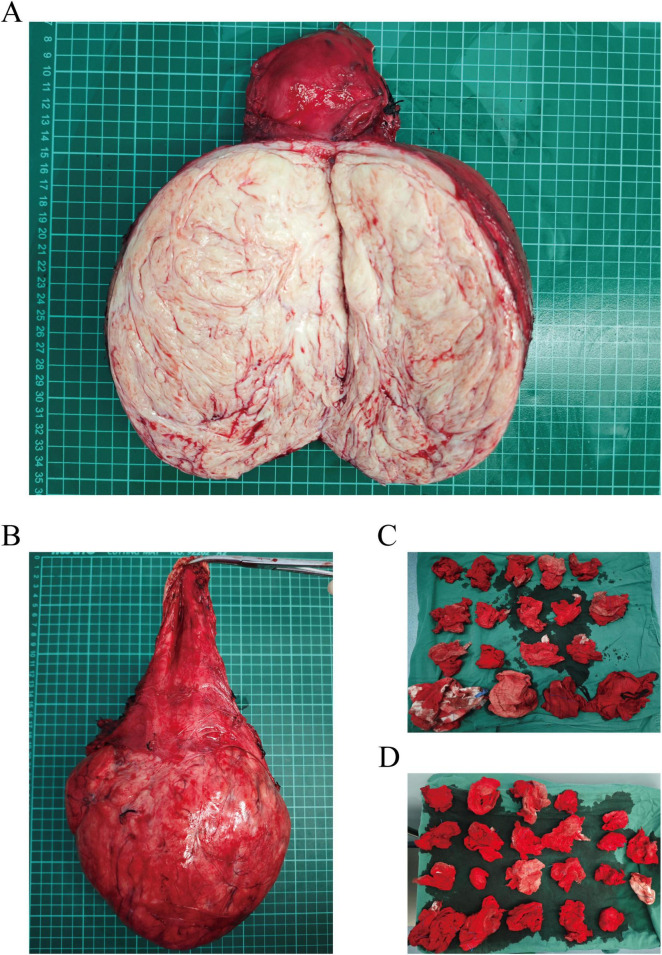
**(A)** Longitudinal section of the cervical myoma specimen. **(B)** Postoperative specimen showing significant elongation of the cervical structure. **(C,D)** Intraoperative assessment of blood loss using gauze evaluation.

Preoperative assessments were conducted to rule out contraindications. Following satisfactory anesthesia, a bilateral ureteral stenting procedure was performed via cystoscopy. The abdominal cavity was accessed through a layered incision, revealing an elevated bladder located between the umbilicus and the pubis (Figure 3A). The bladder was meticulously dissected, and the uterus was retracted. A large cervical myoma, measuring approximately 20 cm in diameter, was identified at the cervical isthmus, with the uterine body slightly enlarged. Injection of posterior pituitary hormone was performed, revealing numerous engorged blood vessels on the surface of the myoma (Figure 3B). The right ovarian ligament and isthmus of the fallopian tube were clamped, cut, and sutured. Profuse bleeding occurred from the cervical myoma, with suboptimal hemostasis achieved through suturing; therefore, ligation of the engorged vessels on the cervical surface was performed, followed by similar treatment on the contralateral side. The bladder was retracted by opening the peritoneum, allowing the bladder to be pushed downward. A longitudinal incision was made along the posterior wall of the cervical myoma, and blunt and sharp dissection was used to separate the capsule, resulting in the excision of the cervical myoma (Figure 3C), which displayed a wide, elongated cervical structure (Figure 3D). A total hysterectomy was performed with resection at the cervical external os (Figures 4A, B). The specimen was sent for pathological examination. Intraoperative blood loss was 800 ml (Figures 4C, D), and 2 units of red blood cell suspension and 400 ml of fresh frozen plasma were transfused. Postoperative treatment included fluid replacement and symptomatic management for anemia, with satisfactory recovery (Figure 1C), leading to a smooth discharge.

### 2.3 Pathological results

Uterine leiomyoma, with locally rich cellularity and active cellular proliferation (Figures 4A, B) showing cervical leiomyoma and pathological specimens sent after total hysterectomy.

## 3 Discussion

Cervical giant leiomyomas are extremely rare in clinical practice, presenting significant challenges in diagnosis and treatment (7). Comprehensive preoperative evaluation and the formulation of an appropriate surgical strategy are particularly crucial (8). Pelvic ultrasound is a commonly used imaging technique for diagnosing leiomyomas; however, when there is suspicion of degeneration, especially considering the potential for sarcomatous transformation, further imaging with CT or MRI can assist in clarifying the nature of the tumor. Both modalities exhibit superior specificity and positive predictive value compared to ultrasound and can somewhat reflect pathological findings (9). Additionally, large leiomyomas can cause displacement of pelvic organs. CT and MRI are better suited to delineate the relationship between the leiomyoma and surrounding pelvic structures, providing vital guidance for the surgeon and potentially reducing intraoperative complications. For typical cervical leiomyomas, T2-weighted MRI images typically reveal low-signal masses with homogeneous enhancement (10). It is common for these leiomyomas to exhibit vascular proliferation, resulting in surrounding flow void phenomena. Uterine leiomyomas have the potential for various forms of degeneration. Transparent degeneration appears as low signal intensity on T2-weighted images, with a lack of enhancement (11). Cystic degeneration typically exhibits high signal intensity on T2-weighted images, but also shows no enhancement. Mucinous degeneration is characterized by marked high signal intensity, with a progressive increase in enhancement (12). On T1-weighted images, red degeneration, which often occurs during pregnancy, presents as high signal intensity, usually due to venous thrombosis (13).

For symptomatic cervical leiomyomas, surgery is the primary treatment approach (14, 15). Currently, laparoscopic myomectomy is mainly indicated for tumors with a diameter of less than 10 cm, while vaginal myomectomy is suitable for submucosal leiomyomas within the cervical canal or smaller leiomyomas adjacent to the cervix (16, 17). However, for patients wishing to preserve fertility, surgical treatment may not be the optimal approach. While uterine artery embolization is a well-established method for treating uterine leiomyomas (18), the rarity of cervical leiomyomas results in relatively few cases of uterine artery embolization (19). It has been reported that the team led by William D alleviated the cervical fibroid burden and relieved clinical symptoms through ovarian artery embolization (20). In this case, the patient presented with a large cervical leiomyoma that had grown rapidly over a short period, raising the possibility of sarcomatous degeneration. Given the patient’s lack of reproductive desire and a comprehensive assessment, an abdominal total hysterectomy was performed. Surgical treatment of large cervical leiomyomas poses significant challenges, necessitating extensive experience and expertise from the surgeon (21). The compression caused by the large leiomyoma may lead to close adhesions with surrounding structures such as the anterior wall of the bladder, the posterior wall of the rectum, and both ureters. Additionally, the size of the leiomyoma may alter the normal positioning of these organs, making it difficult for the surgeon to identify an appropriate entry point during surgery (22). Therefore, preoperative ureteral stent placement was performed to facilitate intraoperative identification of the ureters, which was crucial in preventing iatrogenic ureteral injury (23). In this case, the ureters were significantly displaced and tightly adherent to the uterus due to the compression from the leiomyoma, but the stent placement allowed for accurate identification and successful avoidance of injury during the procedure. The large cervical leiomyoma exerted anterior pressure on the bladder, causing it to closely adhere to the abdominal wall. Preoperatively, careful observation of pelvic MRI results was essential to ascertain the position of the bladder. Special care was taken during the abdominal entry to avoid bladder injury (Figure 3A).

Furthermore, the presence of a large cervical leiomyoma can lead to congestion of the uterine arteries and veins, complicating the surgical procedure (24). The limited operative space and potential structural displacement increase the risk of damage to pelvic organs and make controlling intraoperative bleeding more challenging (25). Intraoperative hemorrhage is a significant risk during cervical leiomyoma surgery, which is related not only to the anatomical proximity of the leiomyoma to major blood vessels but also to the formation of neoangiogenesis within the leiomyoma itself (26). To mitigate the risk of bleeding during surgery, various methods have been developed in clinical practice, including the preoperative use of gonadotropin-releasing hormone agonists(GnRH), intraoperative injection of pituitary gland extracts into the myometrium, and uterine artery embolization (27). Studies have indicated that while uterine artery embolization can effectively reduce intraoperative bleeding during myomectomy and lower recurrence rates, it may adversely affect ovarian blood supply (28), potentially impacting ovarian function in young women; thus, careful consideration is warranted when selecting this approach (29).

Furthermore, due to the potential for large cervical leiomyomas to occupy the entire pelvic cavity, creating significant limitations in the retroperitoneal anatomical space, ligation of the uterine arteries can be extremely challenging, and in some cases, may be impossible (1). In the case presented, the patient’s cervical leiomyoma was excised by longitudinally opening the serosal layer along the posterior wall of the leiomyoma and extracting the tumor from the tail end toward the head side, which allowed for the successful ligation of the uterine vessels. The large cervical leiomyoma exerted significant pressure, leading to notable cervical stretching, where the boundaries of the cervical orifice were flattened (Figure 3C). This made the exposure of the inferior margin of the cervical external os during total hysterectomy particularly challenging, as the uterosacral ligaments originate from the upper posterior aspect of the junction between the body of the uterus and the cervix. The complete resection of the uterus was successfully performed with the uterosacral ligament attachment points serving as the operative boundary.

## 4 Conclusion

Due to the significantly lower incidence of cervical leiomyomas, particularly large cervical leiomyomas, compared to other types of uterine fibroids, there is a lack of clinical experience in patient management. In our report, we aim to share our surgical experience and emphasize the importance of comprehensive preoperative assessment in avoiding surgical complications. We hope that our findings will provide valuable insights for clinical patient management and the formulation of surgical strategies.

## Data Availability

The original contributions presented in this study are included in this article/supplementary material, further inquiries can be directed to the corresponding author.
